# Supplementary material for: The Presence of Caffeic Acid in Cerebrospinal Fluid: Evidence That Dietary Polyphenols Can Cross the Blood-Brain Barrier in Humans
Source: Nutrients. 2020 May 25;12(5):1531. doi: 10.3390/nu12051531 (PMC7284697; doi:10.3390/nu12051531)
Supplement: Supplementary file 1 [file nutrients-12-01531-s001.pdf]

Supplementary Materials

Table S1. Individual results of 3-hydroxyphenyl acetic acid (3HPAA), homovanillic acid (HVA) and caffeic acid (CA) measurement in cerebrospinal fluid and plasma in the subgroup of patients with multiple sclerosis.

Patient Number	Sex	Cerebrospinal Fluid [μmol/L]			Plasma [μmol/L]		
		3HPAA	HVA	CA	3HPAA	HVA	CA
1	F	7.73	0.16	0.03	0.00	0.07	0.03
2	F	0.00	0.15	0.02	0.00	0.07	0.03
3	F	39.15	0.22	0.03	4.12	0.13	0.02
4	F	6.92	0.20	0.01	0.00	0.10	0.03
5	F	7.13	0.29	0.03	0.00	0.07	0.02
6	M	4.50	0.07	0.03	0.00	0.08	0.03
7	F	5.82	0.37	0.03	0.00	0.09	0.03
8	F	3.14	0.27	0.03	0.00	0.07	0.03
9	F	3.01	0.10	0.02	0.00	0.07	0.03
10	F	2.33	0.15	0.00	0.00	0.08	0.03
11	F	2.17	0.23	0.02	0.00	0.13	0.02
12	F	1.19	0.12	0.03	0.00	0.11	0.03
13	M	3.40	0.09	0.03	0.00	0.07	0.03
14	F	17.41	0.16	0.03	1.43	0.10	0.03
15	F	17.96	0.30	0.03	0.93	0.08	0.01

Table 2. Individual results of 3-hydroxyphenyl acetic acid (3HPAA), homovanillic acid (HVA) and caffeic acid (CA) measurement in cerebrospinal fluid and plasma in the subgroup of patients with other neurological disorders.

Patient Number	Sex	Cerebrospinal Fluid [μmol/L]			Plasma [μmol/L]		
		3HPAA	HVA	CA	3HPAA	HVA	CA
1	F	10.19	0.42	0.02	9.93	0.16	0.03
2	F	9.00	0.11	0.02	0.00	0.11	0.03
3	M	2.42	0.47	0.04	0.00	0.16	0.04
4	M	2.63	0.33	0.02	0.00	0.12	0.03
5	M	3.18	0.16	0.02	0.00	0.15	0.04
6	M	19.53	0.23	0.02	0.00	0.10	0.04
7	M	2.38	0.06	0.04	0.00	0.12	0.03
8	F	24.75	0.15	0.02	4.00	0.07	0.04
9	F	3.74	0.27	0.02	0.00	0.11	0.03
10	M	11.59	0.23	0.02	0.00	0.16	0.04
11	M	4.20	0.07	0.02	0.00	0.13	0.03
12	M	2.51	0.17	0.02	0.00	0.05	0.03
13	M	4.97	0.19	0.02	0.00	0.07	0.03
